# Supplementary material for: Antibacterial and Antibiofilm Activities of β-Lapachone by Modulating the Catalase Enzyme
Source: Antibiotics (Basel). 2023 Mar 14;12(3):576. doi: 10.3390/antibiotics12030576 (PMC10044350; doi:10.3390/antibiotics12030576)
Supplement: Supplementary file 1 [file antibiotics-12-00576-s001.zip › antibiotics-2216972-supplementary.pdf]

Supplementary data

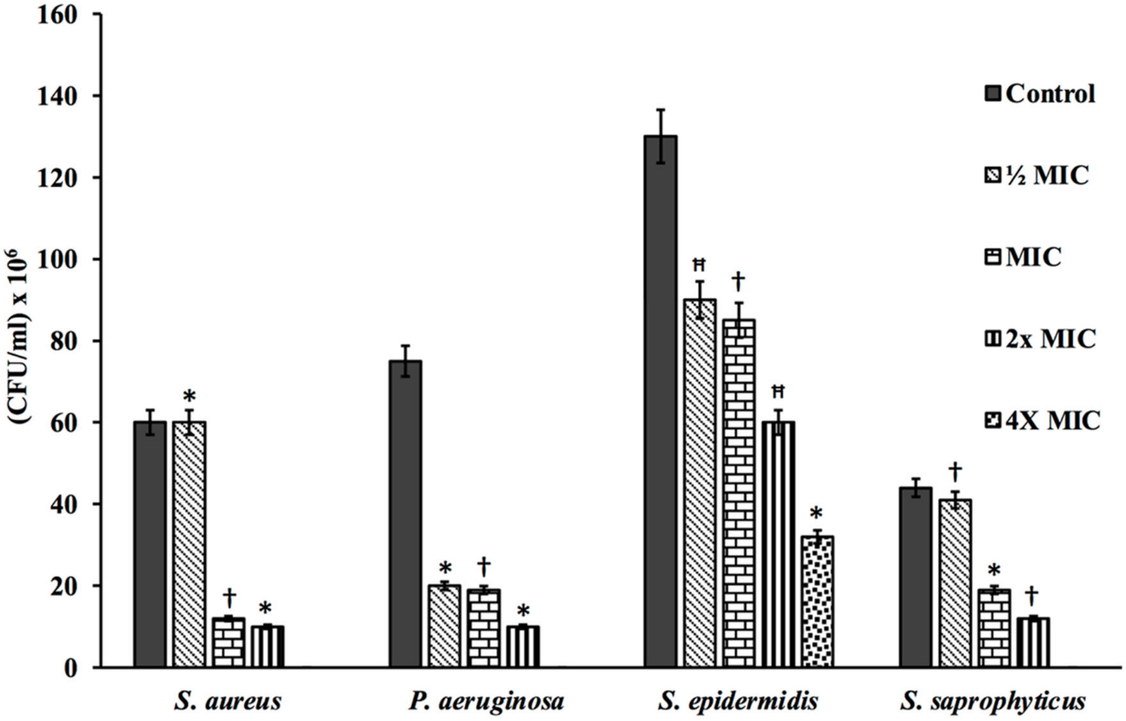

Supplementary Figure S1. CFUs of bacterial strains at different concentrations of β –Lap: The CFUs for each bacterial strains treated with 1/2MIC, MIC, 2xMIC and 4xMIC of β –Lap for 24 hours were enumerated and plotted. The cfu plotted are the average of three technical replicates. *; $p < 0.002$, †; $p < 0.004$, Ħ; $p < 0.007$.

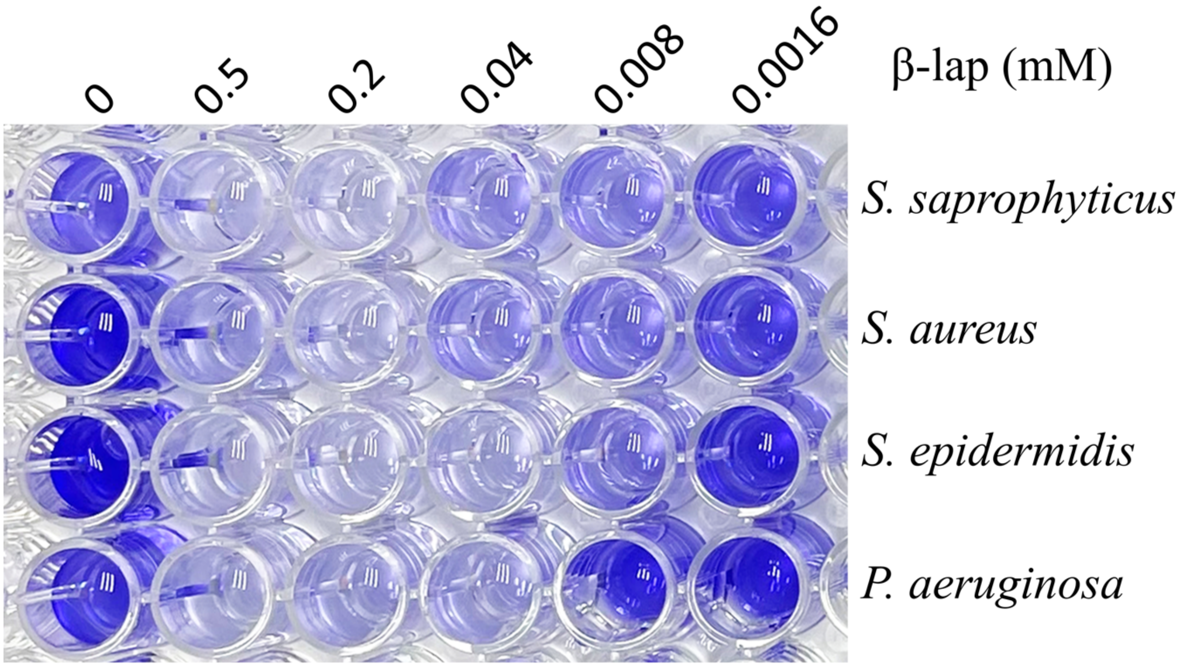

Supplementary Figure S2: β -Lap inhibited the biofilm formation. Image of biofilms formed by S. *saprophyticus*, *S. aureus, S. epidermidis*, and *P. aeruginosa* in presence of different concentrations of β -Lap .

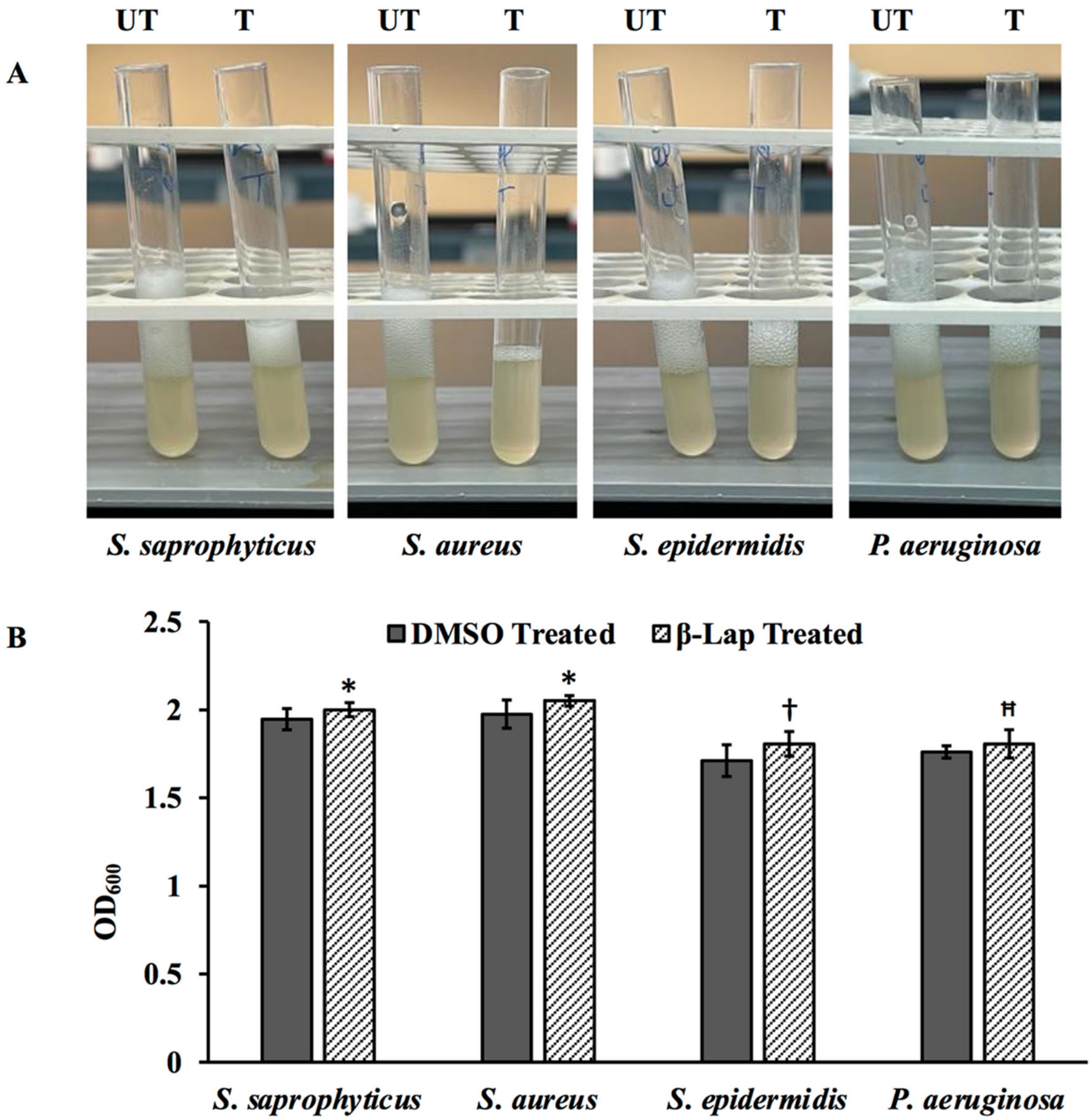

Supplementary Figure S3. Catalase inhibition: A) Out of two equal volume cultures for each strain, one was treated with DMSO and the other with 1/100xMIC of the β -Lap overnight. 200 μL of 3% H2O2 were added to 1m each of DMSO-treated and b-lap-treated cultures of each strain in glass tubes. The tubes were photographed after the air bubbles were formed. B) Before the addition of H2O2, the turbidity of cultures was measured by taking absorbance at 600 nm. UT: DMSO treated, T: β –Lap treated. *; $p < 0.002$, †; $p < 0.004$, Ħ; $p < 0.007$.
